# Error‐Related Brain Activity Indicates Immediate Auto‐Cancellation of Action Slips

**DOI:** 10.1111/psyp.70160

**Published:** 2025-10-07

**Authors:** Roland Pfister, Anna Foerster, Katharina A. Schwarz, Samuel Varga, Marco Steinhauser, Wilfried Kunde

**Affiliations:** ^1^ Experimental Psychology Trier University Trier Germany; ^2^ Institute of Cognitive & Affective Neuroscience Trier University Trier Germany; ^3^ Institute of Psychology University of Wuerzburg Wuerzburg Germany; ^4^ Department of Behavioural and Cognitive Sciences University of Luxembourg Esch‐sur‐Alzette Luxembourg; ^5^ Institute of Psychology Catholic University of Eichstaett‐Ingolstadt Eichstaett Germany

**Keywords:** error processing, executive functions, performance monitoring

## Abstract

The error‐related negativity (ERN) is a classic electrophysiological index of error detection. However, the present study challenges its classical functional interpretation by analyzing the ERN relative to the termination of erroneous actions (response offsets), a behavioral marker of error cancellation. Our findings reveal that the ERN reflects immediate auto‐cancellation of ongoing erroneous behavior. Specifically, our findings corroborate that erroneous responses come with significantly shortened response durations (RDs) compared to correct responses, pointing to an immediate and active cancellation of ongoing motor activity. Crucially, ERN amplitude and latency varied with RDs, indicating that the ERN may reflect not only passive error detection but also the autonomous implementation of corrective behavior. These observations portray human performance monitoring as consisting of two components: a passive component related to detecting action slips, and an active component related to the implementation of behavioral changes. Moreover, these results carry important clinical implications. Abnormal ERN patterns observed in conditions such as obsessive‐compulsive disorder, anxiety, and Parkinson's disease may stem not solely from impaired error detection but from disrupted regulation and cancellation of erroneous actions. By integrating behavioral dynamics with electrophysiological measures, our study highlights the need to reconsider the functional significance of the ERN in both cognitive neuroscience and clinical contexts.

## IMPACT STATEMENT

Electrophysiological activity surrounding an act of error commission has been linked to the monitoring of response conflict or prediction errors. Consequently, models of error processing typically assume that error‐related behavioral adaptations occur only later, during subsequent action episodes. The present findings challenge this view by revealing surprisingly immediate cancellation efforts of aborting ongoing behavior, which directly feed into electrophysiological changes in the immediate aftermath of error commission. This analytical strategy highlights the promise of analyzing electrophysiological activity relative to the offset rather than onset of overt behavior.

## 1 Introduction

To err is human, but every single action slip is critical and needs to be detected as fast as possible. Human neurophysiology has linked fast and efficient error detection to a distinct event‐related potential peaking within only 100 milliseconds after error commission (Renault et al. 1980; Gehring et al. 1993; Falkenstein et al. 1990). This error‐related negativity (ERN) is often seen as the initial step of performance monitoring, preparing the ground for later error correction and strategic adaptation of cognitive processing (Ridderinkhof et al. 2004; Logan and Crump 2010; Danielmeier and Ullsperger 2011; Maier et al. 2011; Wessel and Aron 2017; Dehaene 2018). It also comes with direct clinical implications (Olvet and Hajcak 2008), as abnormal error detection is associated with conditions such as obsessive‐compulsive disorder (Gehring et al. 2000), anxiety and depression (Holmes and Pizzagalli 2008), Parkinson's disease (Beste et al. 2010), as well as schizophrenia (Foti et al. 2012).

While the significance of the ERN in error‐processing has been explored for decades at this point, earlier studies have tended to overlook the behavioral indicators associated with the ERN (see also Kieffaber et al. 2023). Past studies have predominantly employed experimental designs that limit participant responses to discrete key presses, focusing on solely response initiation as opposed to response termination (but see Pfister et al. 2023). This approach blurs the distinction between the onset and conclusion of an action, making it difficult to observe real‐time processes such as error monitoring and correction (Kieffaber et al. 2023). Nonetheless, some approaches tried to address this limitation by either examining “partial errors” in which an error is initiated but a correct response is ultimately given (Vidal et al. 2003; Burle et al. 2008; Masaki et al. 2007; Pailing and Segalowitz 2004; Carbonnell and Falkenstein 2006), or by analyzing continuous movements, such as mouse trajectories (Kieffaber et al. 2016, 2023; Rodríguez‐Fornells et al. 2002). This research suggests that the latency of the ERN is strongly correlated with the peak deceleration of the initiated movement prior to response correction (Kieffaber et al. 2023; Tafuro et al. 2020). In line with these results, recent behavioral observations of immediate error cancellation even for short‐lived keypress actions suggest a fundamentally different reading of the ERN's functional significance (Hochman et al. 2017; Foerster et al. 2022): A distinct ERN signature might in fact originate from immediate efforts to cancel ongoing erroneous movements on the fly rather than reflecting prediction errors or response conflict as previously assumed. However, these behavioral results (e.g., Foerster et al. 2022) were not accompanied by neurophysiological measures to substantiate this inference. Therefore, we hypothesized that stronger behavioral evidence for active cancellation predicts larger ERN amplitudes, shorter ERN latencies, and a distinct contribution of response offset to electrophysiological activity.

**FIGURE 1:**
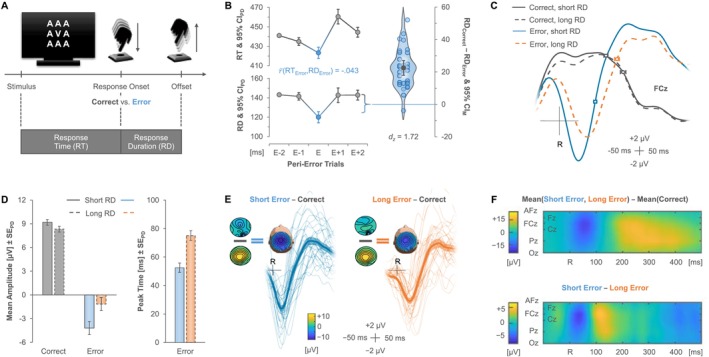
Experimental design and aggregate results. (A) Participants classified a central letter stimulus with left versus right keypresses. Additional irrelevant letter stimuli added visual noise, and a strict response deadline of 600 ms ensured that the task elicited sufficiently many errors for analysis. (B) Response times (RTs) and response durations (RDs) for commission errors (E) and correct peri‐error trials. Error bars indicate 95% confidence intervals for paired differences (CI_PD_) relative to the immediately preceding response. Commission errors came with systematically reduced RDs, indicating active error cancellation. (C) Event‐related potentials at electrode site FCz, time‐locked to response onset (R). Data for correct responses and errors were split at their median RD across trials to investigate the impact of error cancellation on the error‐related negativity (ERN), with response offsets ± standard error shown as point overlays. (D) Mean amplitudes (left; time window: [32 ms, 82 ms] post‐error) and peak times (right) at electrode FCz with corresponding standard errors of paired differences (SE_PD_; see the Supporting Information for multi‐electrode results). (E) Individual ERN waveforms for short and long errors relative to correct responses, including their spatial topography. (F) Temporal evolution of the ERN and the difference between short and long errors across the vertex electrodes.

We probed for this possibility by measuring electroencephalographic (EEG) activity in an effective sample of 30 healthy participants while they performed an error‐eliciting choice‐response task. This task required speeded left versus right responses to target letters that were displayed on screen (Figure 1A; see the Supporting Information for details). Irrelevant letter stimuli surrounded the target; they were not mapped to any response but yielded visual noise, and a strict response deadline ensured that participants would commit sufficiently many errors. For each response, we measured its onset (response time, RT), and crucially its offset, to determine response duration (RD; i.e., the time between pressing and releasing the key, Pfister et al. 2023).

Both measures come with a unique pattern surrounding erroneous actions. RTs decrease prior to error commission (pre‐error speeding), and they markedly increase following an error (post‐error slowing; Dudschig and Jentzsch 2009; Jackson and Balota 2012; Pfister and Foerster 2022). The interplay of pre‐error speeding and post‐error slowing has been suggested to reflect a shift in decision criteria with impulsive responding immediately before error commission and cautious responding following error commission (Jentzsch and Dudschig 2009; Danielmeier and Ullsperger 2011; Hoffmann and Beste 2015; Steinhauser et al. 2017). Post‐error slowing further captures attentional distraction (Notebaert et al. 2009), and it is especially pronounced when erroneous responses cannot be corrected, indicating sustained processing of potential correction responses (P. M. A. Rabbitt 1966; Crump and Logan 2013).

Recent observations pointed towards a diverging pattern for RDs, with particularly short RDs for erroneous responses as compared to all correct responses surrounding the error (Hochman et al. 2017; Foerster et al. 2022). This pattern is stable across the entire RT distribution and cannot be explained in terms of other biomechanical differences such as reduced force of erroneous responses (P. Rabbitt 1978; Foerster et al. 2022). Further, shortened RDs for erroneous responses arose mainly after a response reached its peak force. Together, the findings for RDs therefore indicate immediate and active cancellation or termination of ongoing motor activity. We thus assessed electrophysiological activity as a function of RD to explore the neurophysiological underpinnings of error cancellation. To this end, we split the data into responses with short versus long RDs, predicting a particularly strong impact of error cancellation for short durations. We then followed up on this coarse‐grained procedure by computing a residue‐iteration decomposition (RIDE) analysis to elucidate the unique contribution of response offsets to ongoing electrophysiological activity.

## 2 Materials and Methods

### 2.1 Power Analysis, Sample Size, and Participant Demographics

The ERN is a highly reliable component of the event‐related potential (ERP) that is readily observed in single‐subject data. Similarly, large error cancellation effects on RDs have recently been reported (*d*_*z*_ = 1.34; Foerster et al. 2022). We therefore based our sample size on a conservative power analysis assuming a generic medium effect size of *d*_*z*_ = 0.50 (power 1‐*β* = 0.80, *α* = 0.05), suggesting a sample size of 34 participants. This sample size ensures a power of 1‐*β* > 0.99 for detecting previous RD effects. We preregistered this target sample size on the Aspredicted platform (https://aspredicted.org/LR9_JJL).

An initial sample of *N* = 34 participants was recruited through advertisements in the local participant recruitment system. Their mean age was 28.53 years (range: 20–64 years). Nine participants self‐identified as male, 25 as female, 3 as left‐handed, 29 as right‐handed, and 2 as ambidextrous. All participants reported normal or corrected‐to‐normal visual acuity. The study was conducted in accordance with the guidelines of the ethics committee of the Institute of Psychology, University of Würzburg, Germany. We did not seek individual approval for this study protocol for using a mundane choice response task with healthy participants. All participants provided written informed consent before participation.

The data of four participants had to be excluded from the analysis: Three datasets did not match our pre‐determined inclusion criteria of at least 10 usable trials per condition, and one dataset had missing data in the electrooculogram (EOG) readings, which did not allow for meaningful correction of blink and eye‐movement artifacts. The effective sample size of 30 participants still came with a power of 1‐*β* > 0.99 for standard ERN analysis and behavioral error cancellation effects alike.

### 2.2 Apparatus and Electrophysiological Recording

Participants completed the task in an electrically shielded chamber. Stimuli appeared on a 17“ monitor (75 Hz), and participants responded with their left and right index fingers on the two lateral keys of a PST Chronos device (Psychology Software Tools; Sharpsburg, PA, USA; the first author thanks PST for sponsoring the device as part of their E‐Prime Challenge 2019). This setup allowed us to record response onsets and offsets with ≤ 1 millisecond precision, thus yielding precise estimates of the corresponding RDs.

We recorded EEG activity with a BrainVision QuickAmp amplifier with 32 active electrodes (actiCAP; Brain Products, Germany). Electrode sites were arranged according to the international 10–20 system, with electrodes placed at FP1, FP2, F7, F3, Fz, F4, F8, FCz, FC1, FC2, AFz, T7, C3, Cz, C4, T8, TP9, CP1, CP2, TP10, P7, P3, Pz, P4, P8, PO9, O1, Oz, O2, PO10, as well as the left and right mastoids (M1, M2). The EEG signal was recorded using average reference with a sampling rate of 1000 Hz and impedances < 10 kΩ.

Additional passive electrodes above and below the left eye, as well as at the outer canthi of both eyes tracked the vertical and horizontal electrooculogram (EOG) to control for eye movements.

### 2.3 Task

Participants responded to one of four target letters on every trial with a left versus right keypress. The letters *R* and *N* mapped to one response, whereas *V* and *K* mapped to the other response, with counterbalanced mapping of the two letter pairs to responses across participants. The target letter appeared centrally in a grid of 3 × 3 characters. The other eight characters displayed one of eight irrelevant letters that did not map onto any response (i.e., *O*, *W*, *X*, *U*, *Z*, *Y*, *H*, or *A*; all eight irrelevant letters had the same identity on each trial). We instructed participants to ignore the irrelevant letters and inserted these stimuli only to elicit errors through perceptual noise. All letters were displayed in white font on a black background. The combination of four target letters and eight irrelevant letters, respectively, resulted in 32 individual stimulus constellations. In an initial practice block, participants went through a random order of these combinations. In the following 19 experimental blocks, each combination appeared twice at a random position in the block, resulting in 64 trials per block. Blocks were separated by self‐paced breaks, during which a written message encouraged participants to respond as fast and accurately as possible throughout the experiment.

The first trial of each block started with a fixation of 750 ms. Then the target letter appeared for a maximum of 600 ms, which disappeared when participants responded before that deadline. Participants immediately received feedback about the accuracy of each response for 1000 ms in the practice block (translated from German: “Good!” in green font for correct responses; “Wrong!” in red font for incorrect keypresses and “Too slow!” in red font if they omitted their response). Participants only received feedback for omission errors in experimental blocks, but no feedback for correct responses or commission errors. However, we provided aggregate feedback about the mean correct RT, the number of incorrect keypresses, and the number of omission errors after each block. Afterward, a white fixation cross appeared centrally for 500 ms, and then the screen went blank for 1000 ms. We continued to collect key presses and releases after target presentation to measure RD and to identify and exclude trials with late additional responses (labeled miscellaneous errors in the following).

### 2.4 Data Preprocessing

All preprocessing and analysis steps were implemented via custom R scripts (using the packages *ez*, *doBy*, *MBESS*, and *schoRsch*) as well as custom Matlab code that utilized the FieldTrip toolbox (Oostenveld et al. 2010). All scripts are available on the Open Science Framework (https://osf.io/4vncf/).

For the behavioral data, we first removed all trials of the practice block. We then searched through the data and selected all trials with commission errors (10.09% of all trials; miscellaneous errors with anticipative responses or more than one keypress: 0.34%; omission errors: 8.64%). From these trials, we selected only those commission errors with two preceding correct trials and two following correct trials. We departed from our pre‐registered plans of using only ±1 correct peri‐error trials for the behavioral analysis to be able to compute pre‐error speeding and post‐error slowing relative to close peri‐error trials (Pfister and Foerster 2022; using the trial selection of the EEG analysis produced virtually identical results for all following RD analyses):

$$\begin{array}{c} \mathrm{Pre}-\text{error speeding}=\frac{\left({\mathrm{RT}}_{E-2}+{\mathrm{RT}}_{E+2}\right)}{2}-{\mathrm{RT}}_{E-1}\\ \text{Post}-\text{error slowing}={\mathrm{RT}}_{E+1}-\frac{\left({\mathrm{RT}}_{E-2}+{\mathrm{RT}}_{E+2}\right)}{2} \end{array}$$

We screened the data for outliers as defined by RD deviating more than 2.5 standard deviations (SDs) from the corresponding cell mean, computed separately for correct and erroneous responses of each participant (1.34%). To retain a balanced dataset, we removed each chunk of error and its accompanying ±2 correct peri‐error trials when detecting at least one outlier in the sequence, removing 5.96% of the error sequences.

For the remaining data, we determined for each trial whether its RD was shorter or longer than the condition median, again calculated separately for correct and erroneous responses of each participant. RDs exactly matching the median were assigned to the slower bin.

EEG preprocessing used all error trials that came with at least one preceding correct trial. We first read trial segments of 1200 ms around the event of interest (stimulus onset, response onset, response offset) and baseline‐corrected each epoch (stimulus‐locked baseline: [−100 ms, 0 ms]; response‐onset baseline: [−150 ms, −50 ms]; response‐offset baseline: [−300 ms, −200 ms]). We then applied a band‐stop filter to remove line noise ([47.5 Hz, 52.5 Hz]) using FieldTrip's 4th order forward‐backward Butterworth infinite impulse response (IIR) filter, and subjected the resulting data to the artifact detection routines of FieldTrip based on the average *z*‐value of the signal amplitude (Hilbert envelope) across electrodes (jump artifacts: cutoff = 20; muscle artifacts: cutoff = 8 with band‐pass filter at [110 Hz, 140 Hz]). Epochs containing at least one artifact were removed from the data (12.56% of the trials).

To avoid biases due to different trial numbers after artifact rejection, we assessed the frequency of the remaining short and long error trials in each dataset and removed trials with RDs closest to the median RD of the condition with more data points until both conditions had identical trial numbers (resulting in 19.43 remaining error trials per RD condition on average).

The final selection of trials then entered a correction for blink and eye movement artifacts based on independent component analysis (ICA). After ICA decomposition, we removed components that correlated with either EOG channel (threshold: *r* = 0.40) and recomposed the data. We then re‐referenced the data to linked mastoids, re‐applied the corresponding baseline corrections, and filtered the final data at 0.1 Hz high‐pass and 20 Hz low‐pass (both 4th order).

**FIGURE 2:**
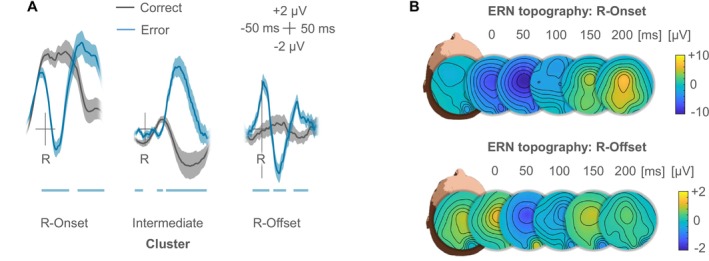
Single‐trial analyses for erroneous and correct responses. (A) Residue‐iteration decomposition (RIDE) of the signal at electrode FCz into three distinct clusters, with marked error‐related negativity (ERN) in the response onset cluster and the response offset cluster (*R* = response). Shaded areas indicate standard errors of paired differences between correct and erroneous responses, whereas horizontal bars show significant differences as indicated by permutation tests. Intriguingly, only errors elicited reliable activity in the response offset cluster, reinforcing the active cancellation account. (B) Topographies for the difference wave (Error − Correct) for both clusters that contributed to the ERN (R‐Onset, R‐Offset). Timestamps mark the beginning of each time window of 50 ms.

The described preprocessing algorithm applied to all main analyses. Adjustments of these general procedures were only implemented for analyses using RIDE analyses and for analyses assessing the lateralized readiness potential (LRP). For our initial RIDE analysis, we collapsed the data across RD bins, because this single‐trial analysis leverages the trial‐by‐trial variability of different signal components (here: response onset versus response offset) and therefore does not require any prior binning. This analysis was complemented by separate RIDE analyses of short and long errors to assess amplitude effects in addition to the latency focus of the initial RIDE analysis. For both applications of the RIDE algorithm we tested whether a decomposition into 3 clusters was warranted over a solution with only two clusters (R‐Onset and R‐Offset without an R‐Intermediate cluster in between; with *R* = response). Even though the crucial observation of a distinct ERN response in the R‐Offset cluster emerged in 2‐ and 3‐cluster solution alike, the intermediate cluster turned out to capture the distinct P_E_ component of the ERP (e.g., Nieuwenhuis et al. 2001; Overbeek et al. 2005), so that we chose to implement all three clusters (see Figure 2; the P_E_ was located in the R‐Offset cluster for two‐cluster solutions). We further ensured that RDs introduced a temporal jitter with sufficiently meaningful variation as compared to response times. This was indeed the case as suggested by analyses of Coefficients of Variation (CV) with CV_RD_ even exceeding CV_RT_ for correct responses and errors alike, *t*(33) > 4.55, *ps* < 0.001, *d*_*z*_ > 0.83. LRP analysis additionally split the data according to the actual response (left vs. right) in order to compute the LRP. We restricted this analysis to a subset of 15 participants with at least 15 trials per error type and did not enforce equal trial numbers per condition to be able to compute meaningful LRP statistics. We verified that the subset of participants showed similar ERP responses as the full sample to establish that potential LRP results do not reflect specific properties of the selected sub‐set.

### 2.5 Simulations

**FIGURE 3:**
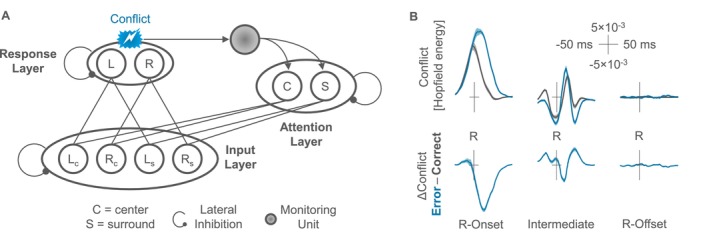
Model architecture and simulation results. (A) The model comprised three layers in a neural network. Excitatory connections were effective between layers, whereas individual units within the same level inhibited each other. Separate units coded for the left and right response (L vs. R) based on input in the central target (C) and the surrounding irrelevant stimuli (S). The figure shows only one surround unit in the input layer and the attention layer for simplicity, though simulations used two independent surround units in each layer. Conflict was computed as the activation product of both units in the response layer (Hopfield energy). Responses were registered whenever the activation of one unit surpassed a threshold value, whereas response offset was registered when activation dropped below this threshold. (B) Residue‐iteration decomposition (RIDE) of the simulated conflict into three distinct clusters relative to the response. Shaded areas indicate the standard error of paired differences for individual data points based on 34 simulated datasets. The simulation results yielded a clear ERN‐like signature in the R‐Onset cluster, whereas the network did not produce any systematic activity in the R‐Offset cluster.

Finally, we implemented a computational model to test whether the observed ERN signature locked to response‐offsets can be explained by conflict‐monitoring theories of the ERN (Carter et al. 1998; Botvinick et al. 2001; Gehring and Fencsik 2001; Yeung et al. 2004). Conflict‐monitoring accounts explain the ERN as reflecting conflict that is triggered by the parallel activation of competing action plans. In this computational formulation, conflict is proportional to the activation product of two competing response nodes in a recurrent neural network (the Hopfield energy; Botvinick et al. 2001; Yeung et al. 2004). Simulations with this model provide an elegant account for the ERN signature being tied to the preparation and onset of erroneous actions. To determine whether they can also account for offset‐locked activity, we implemented a variant of the conflict monitoring model (Yeung et al. 2004) that we adapted to the present task. Figure 3 provides a schematic of the model architecture.

## 3 Results

### 3.1 Main Findings

A mean error rate of 10.3% (SD = 4.1%) for commission errors confirmed that the task succeeded in eliciting sufficiently many commission errors for meaningful analysis. All other potential errors such as response omissions or multiple responses within a single trial accounted for another 11.2% of the trials (SD = 7.4%).

Figure 1B shows both behavioral measures, i.e., RDs and RTs, for erroneous responses, the two correct responses preceding an error, and the two correct responses following this error. A repeated‐measures analysis of variance (ANOVA) on mean RDs yielded a significant effect of trial sequence (E−2, E−1, E, E+1, E+2; E = error), *F*(4, 116) = 71.01, *p* < 0.001, *n*_*p*_^2^ = 0.71 (Greenhouse–Geisser corrected for violation of the sphericity assumption; *ε* = 0.48). Error cancellation was evident in consistently shorter RDs for errors than for correct responses (Figure 1B; 120 ms vs. 142 ms), *t*(29) = 10.12, *p* < 0.001, Δ = 22 ms, 95% CI_Δ_ = [18 ms, 27 ms], *d*_*z*_ = 1.85, 95% CI_SM_ = [1.25, 2.44] (CI_SM_ = confidence interval for standardized means). A second ANOVA on RTs also suggested pronounced differences across the trial sequence, *F*(4, 116) = 48.79, *p* < 0.001, *n*_*p*_^2^ = 0.63 (*ε* = 0.76), with significant pre‐error speeding (RT_E±2_—RT_E‐1_; 443 ms vs. 435 ms), *t*(29) = 3.92, *p* = 0.001, Δ = 7.88 ms, 95% CI_Δ_ = [3.77 ms, 12.00 ms], *d*_*z*_ = 0.71, 95% CI_SM_ = [0.31, 1.11], and post‐error slowing (RT_E‐1_—RT_E±2_; 460 ms vs. 443 ms), *t*(29) = 7.29, *p* < 0.001, Δ = 17.71 ms, 95% CI_Δ_ = [12.74 ms, 22.68 ms], *d*_*z*_ = 1.33, 95% CI_SM_ = [0.83, 1.82]. RD effects were independent of any RT differences in within‐participant analyses as well as across participants (see the Section 3.2 below). These observations thus reinforce the interpretation of error‐related RD effects as active error cancellation (Foerster et al. 2022; see Figure S1 and Table S1 in the Supporting Information for converging evidence from lateralized readiness potentials).

We thus split the data of each participant into responses with RDs shorter and longer than the individual median RD—computed separately for errors and correct responses—and tested whether short versus long errors would give rise to different ERN signatures in a first step. This was indeed the case, with consistently larger ERN amplitudes for short errors (see Figure 1C–E; −4.18 μV vs. −1.16 μV), *t*(29) = 3.66, *p* = 0.001, Δ = 3.02 μV, 95% CI_Δ_ = [1.33 μV, 4.71 μV], *d*_*z*_ = 0.67, 95% CI_SM_ = [0.27, 1.06] (determined at electrode site FCz in the range [32 ms, 82 ms], see Figures S2 and S3 and Tables S2–S7 in the Supporting Information for full multi‐electrode results). All but one of the participants still showed a visible ERN signature in both conditions; restricting the analysis to these individuals further indicated that short errors came with a substantially earlier ERN peak than long errors (52 ms vs. 75 ms), *t*(28) = 6.67, *p* < 0.001, Δ = 23 ms, 95% CI_Δ_ = [16 ms, 30 ms], *d*_*z*_ = 1.24, 95% CI_SM_ = [0.75, 1.72] (see Tables S8 and S9 for full statistics). The spatiotemporal evolution of the observed ERN followed its traditional frontocentral scalp distribution along the vertex (Renault et al. 1980; see Figure 1F). More negative voltages for short as compared to long errors appeared across the central midline electrodes throughout the ERN range, followed by a short period of increased error positivity from 100 to 150 ms after error commission (see also Tables S10 and S11 in the Supporting Information for converging evidence from cluster‐based permutation tests).

As the cancellation account predicts the ERN to be linked specifically to the individual offset of a response, we further performed residue iteration decomposition (RIDE) analyses on the single‐trial EEG data (Ouyang et al. 2015). We configured the algorithm to isolate three distinct clusters related to the onset of the response (R‐Onset cluster), the offset of the response (R‐Offset cluster), separated by an intermediate cluster (R‐Intermediate). We further implemented the algorithm in a maximally conservative fashion by ascribing shared variance of the R‐Onset and the R‐Offset clusters to the former cluster. Despite this conservative approach, RIDE analysis still revealed two distinct ERN‐like signatures related to response onset (correct: 9.13 μV; error: −1.04 μV), *t*(29) = 10.48, *p* < 0.001, Δ = 10.16 μV, 95% CI_Δ_ = [8.18 μV, 12.15 μV], *d*_*z*_ = 1.91, 95% CI_SM_ = [1.30, 2.51], and to response offset (correct: 0.19 μV; error: −1.33 μV), *t*(29) = 5.12, *p* < 0.001, Δ = 1.51 μV, 95% CI_Δ_ = [0.91 μV, 2.12 μV], *d*_*z*_ = 0.93, 95% CI_SM_ = [0.50, 1.36], respectively (Figure 2a; using a time window of [50 ms, 100 ms] post‐response to account for the later time course of the ERN in the R‐Offset cluster). These findings extend the condition‐level observations to single‐trial variability of RDs, while follow‐up analyses suggested that the R‐Onset and the R‐Offset Cluster contribute independently to the observed amplitude differences between errors with short and long RDs (see Figures S4 and S5 and Tables S12 and S13 in the Supporting Information for full statistics). The ERN thus cannot be explained by response onsets alone but is critically dependent on the termination of erroneous motor activity.

### 3.2 Additional Behavioral Results

For the behavioral results reported above, we computed error cancellation effects as RD_correct_—RD_error_, with RD_correct_ being the average of all four correct peri‐error trials (E−2, E−1, E+1, E+2; E = error trial, RD = response duration). Alternative computational methods yielded the same pattern of results, specifically when computing cancellation only against the immediately preceding and following peri‐error trial (120 ms vs. 142 ms), *t*(29) = 9.70, *p* < 0.001, Δ = 22 ms, 95% CI_Δ_ = [17 ms, 27 ms], *d*_*z*_ = 1.77, 95% CI_SM_ = [1.19, 2.34]. The same was true when restricting the analysis to those trials that remained after EEG artifact rejection (121 ms vs. 143 ms), *t*(29) = 9.71, *p* < 0.001, Δ = 22 ms, 95% CI_Δ_ = [18 ms, 27 ms], *d*_*z*_ = 1.77, 95% CI_SM_ = [1.19, 2.34].

Crucially, RD and RT were statistically independent of one another, suggesting that RD effects do not reflect differences in response initiation as captured by RT (nor do they depend on peak force; Foerster et al. 2022; Gehring and Fencsik 1999). Particularly, the average across‐trial correlation of RD and RT (re‐transformed from averaged Fisher‐Z transformed correlations) was $\bar{r}$ = −0.043 for errors and $\bar{r}$ = 0.034 for correct responses. Pre‐error speeding and post‐error slowing were correlated on a trial‐by‐trial level, $\bar{r}$ = 0.421, but neither RT‐based measure correlated with RD effects of error cancellation |$\bar{r}$| < 0.038. No significant across‐participants correlations emerged between pre‐error speeding, post‐error slowing and error cancellation either, |*r*| < 0.113.

### 3.3 Simulation Results

The model included three layers, closely mirroring the architecture used in previous work (Yeung et al. 2004). The only major difference to this previous model was that we only fed noise into units that responded to surrounding irrelevant stimuli to account for the fact that the irrelevant letters were not mapped to any response in the present design, whereas the original model was built for a design that included congruent and incongruent distractor stimuli, which also activated particular input units directly. Repeating the simulation with a classical parameterization of the model including response‐congruent and response‐incongruent trials reproduced the reported data pattern, however.

An input layer included four units coding for the presence of stimuli that were associated with either a left‐hand or right‐hand response in the center of the display (C) or among the surrounding stimuli (S). Units in the input layer projected to a response layer that included one unit for the left‐hand response (L) and one unit for the right‐hand response (R). The network calculated response conflict as measured via the parallel activation of both response units (Hopfield energy). This measure fed into a monitoring unit that was connected to an attention layer with two units that allocated attention to the center or the surround, respectively.

We simulated 34 datasets of 1248 trials each, as implemented in the actual experiment. On each trial, we simulated the network for 50 cycles, including 3 preparatory cycles, with each cycle corresponding to 16 ms of actual time. The net input to all units was set to 0 at the beginning of each run. Both response units then received an input of ext_*i*_ = 0.03 during each of the three preparatory cycles, scaled by a constant of extscale = 0.4 applied to all external inputs. Then, stimulus onset was defined as providing an additional external input of ext_*i*_ = 0.15 to one of the two center units of the input layer. For each cycle, we computed the net input to each unit *i* to determine its activation. In addition to external inputs, the internal input was summed across all connected units *j*, weighted by the connection strength *w*_*ij*_ and a scaling parameter *intscale*_*j*_:

$${\text{input}}_{i}=\left({\mathrm{ext}}_{i}\cdot \text{extscale}\right)+\sum_{j}{\text{activation}}_{j}\cdot w_{\mathrm{ij}}\cdot {\text{intscale}}_{j}+\varepsilon$$

The connection weights were *w*_*ij*_ = 1.5 for feedforward excitatory connections from input units to response units, *w*_*ij*_ = 2.0 for bidirectional excitatory connections between input units and attention units. Pairwise inhibitory connections between all individual units of the same layer were *w*_*ij*_ = −2.0 in the input layer, *w*_*ij*_ = −3.0 in the response layer, and *w*_*ij*_ = −1.0 in the attention layer. The scaling parameter was intscale_*j*_ = 0.08 for excitatory connections and intscale_*j*_ = 0.12 for inhibitory connections. Random noise *ε* was added to all units on each cycle:

$$\varepsilon \sim N\left(0,0.035\right)$$

Crucially, the model captured conflict in terms of the Hopfield energy of the response layer on every cycle with an additional minimum bound at 0:

$$\text{energy}=\max\left(0,-2\cdot \left({\text{activation}}_{L}\cdot {\text{activation}}_{R}\cdot w_{\mathrm{ij}}\right)\right)$$

Because both response units receive (noisy) activation in each cycle, conflict arises for correct and erroneous responses alike. The conflict signal is considerably stronger on error trials, however, and the difference between the response‐locked conflict signal between erroneous and correct responses fits the ERN.

The model further included conflict adaptation as a function of conflict in previous episodes (Botvinick et al. 2001; Carter et al. 1998). To this end, we computed the total energy *E* for each cycle as the sum across all timepoints of the present trial:

$$E_{t}=\sum_{k=1}^{t}{\text{energy}}_{k}$$

The total energy computed in one cycle directly fed into the attention layer in terms of external input on the center unit in the following cycle:

$${\mathrm{ext}}_{C}=\lambda \cdot {\mathrm{ext}}_{C\left(t-1\right)}+\left(1-\lambda \right)\cdot \left(\alpha \cdot E_{\left(\text{cycle}-1\right)}+\beta \right)$$

with ext_*C*(*t‐1*)_ being the external input to this unit in the preceding cycle and *E*_*t‐1*_ being the total energy of the response layer in the previous cycle, scaled with scaling values of previous instantiations of this model (*λ* = 0.5, *α* = 4.41, *β* = 1.08; Yeung et al. 2004). External input to the center unit was further constrained at 1 ≤ ext_*C*_ ≤ 3. We used two surround attention units that both received input as a direct function of the external input to the center unit:

$${\mathrm{ext}}_{S}=0.5\cdot \left(3-{\mathrm{ext}}_{C}\right)$$

The activation of unit *i* depended on input_*i*_ and a decay function:

$$\begin{array}{c} \Delta {\text{activation}}_{i}\\ \;=\left({\text{input}}_{i}\cdot \left({\text{activation}}_{\text{crit}}-{\text{activation}}_{i}\right)\right)-\left(\text{decay}\cdot \left({\text{activation}}_{i}-{\text{activation}}_{\text{rest}}\right)\right) \end{array}$$

with activation_crit_ = activation_max_ = 1.0 for input_*i*_ > 0 and activation_crit_ = activation_min_ = −0.2 otherwise. The boundaries of activation were activation_min_ and activation_max_, respectively, and the decay parameter was set to 0.1 throughout. Response onset was determined whenever activation_*L*_ or activation_*R*_ exceeded a threshold of 0.18 for the first time in a trial, whereas response offset (and thus RD) was determined when the activation of the unit went below this threshold again. Input to the model was stopped at a random time after response onset sampled from a normal distribution (*μ* = 6 cycles, *σ* = 0.5), with a lower bound at 5 cycles.

Operationalizing RDs as the time that a response is activated at suprathreshold level predicts shorter RDs for errors, because errors receive strong lateral inhibition from the concurrently activated correct unit in the response layer. Whether this intuitive property of the model would translate into a distinct ERN signature locked to the offset of a response was a core question of the conducted simulations. The corresponding data yielded clear evidence against this possibility as shown in Figure 3.

## 4 Discussion

The present experiment carried two main discoveries. First, the observation of systematically increased ERN amplitudes for responses of short relative to long duration and, second, distinct ERN responses for the response onset cluster and for the response offset cluster of the RIDE analyses. These observations characterize the ERN as comprising at least two functionally dissociable components, one related to the onset of erroneous behavior and one related to its termination. Whereas the first, passive component is well explained by previous mechanistic accounts of the ERN in terms of conflict caused by the parallel activation of correct and erroneous action plans (Carter et al. 1998; Botvinick et al. 2001; Gehring and Fencsik 2001; Yeung et al. 2004) and in terms of reward prediction errors (Holroyd and Coles 2002), the second, active component is a unique index of active error cancellation. Indeed, a crucial question is whether a response‐offset‐locked component of the ERP would be predicted by common accounts of the ERN. This is not the case. Reinforcement‐learning accounts propose that the ERN is linked to a reward prediction error that occurs when an erroneous response leads to a decrease in the expected action outcome, so that the ERN is “not directly tied to error commission” (Holroyd and Coles 2002). Such an account cannot explain the present results, because neither response duration nor response offset is informative regarding the action outcome. Whether the two components emerge from distinct neuroanatomical sources is an open question (Dehaene et al. 1994; Carter et al. 1998; Gehring and Fencsik 2001). This architecture highlights a critical role for immediate auto‐cancellation of erroneous actions, indicating that the cancellation of erroneous behavior is an instant, hard‐wired corollary of error detection. Error cancellation might further prepare the stage for efficient error correction by performing the originally intended action (P. M. A. Rabbitt 1966; Rodríguez‐Fornells et al. 2002; Fiehler et al. 2005; Bode and Stahl 2014; Roger et al. 2014; Beatty et al. 2021).

Viewing the ERN as an index of error cancellation elegantly integrates a range of classic findings such as higher ERN responses with increasing error significance (Hajcak et al. 2005; Maier and Steinhauser 2013), as agents should be particularly eager to cancel costly errors. Revisiting these classic findings with a focus on error cancellation thus promises to solve some of the long‐standing mysteries surrounding this prominent neurophysiological correlate of human error processing (Cavanagh and Frank 2014; Gehring et al. 2018). This focus also holds potential for refining clinical applications by assessing whether abnormal ERN signatures mainly derive from changes in terms of error detection or whether they relate to abnormal error cancellation instead, thus promising a stronger grasp on the neurophysiological operations underlying these psychiatric conditions. Patients with Parkinson's disease, for instance, show reduced ERN responses relative to control participants, which has typically been seen as indicating deficient performance monitoring (Falkenstein et al. 2001; Stemmer et al. 2007; Beste et al. 2009, 2010). The present findings indicate that these patients may have residual or even intact error detection while not engaging in active cancellation of erroneous motor action. This hypothesis would resonate with common motor‐related symptoms associated with Parkinson's disease (Desmurget et al. 2004). Similarly, larger ERN responses for OCD patients relative to control participants may indicate a strong impulse to cancel erroneous motor activity rather than, or in addition to, overactive monitoring (Gehring et al. 2000; Endrass et al. 2008). Similar hypotheses present themselves for many other clinical conditions that are commonly seen as involving abnormal performance monitoring (Olvet and Hajcak 2008).

On a broader scale, the present observations highlight the promise of systematically assessing the termination of ongoing actions. Behavioral measures such as RDs and electrophysiological approaches such as analyses related to response offset can be easily implemented in a wide variety of tasks in healthy participants and clinical populations alike (Morein‐Zamir et al. 2004; Pfister et al. 2023). Moreover, sophisticated experimental methodology has been developed to assess the termination of action plans that have not yet been executed (Verbruggen and Logan 2009; Verbruggen et al. 2019). Adapting such methodology to study error cancellation holds promise to arrive at a detailed understanding of this intriguing process.

## Author Contributions

**Roland Pfister:** conceptualization, funding acquisition, writing – original draft, methodology, validation, visualization, writing – review and editing, software, formal analysis, data curation, supervision, resources, investigation. **Anna Foerster:** conceptualization, methodology, project administration, writing – review and editing, investigation. **Katharina A. Schwarz:** writing – original draft, writing – review and editing. **Samuel Varga:** writing – review and editing. **Marco Steinhauser:** methodology, writing – review and editing. **Wilfried Kunde:** resources, methodology, writing – review and editing.

## Conflicts of Interest

The authors declare no conflicts of interest.

## Supporting information


**Figure S1:** Lateralized readiness potentials (LRPs), time‐locked to response onset and response offset, respectively.
**Figure S2:** Event‐related potentials (ERPs), locked to response onset (R).
**Figure S3:** Event‐related potentials (ERPs), locked to response offset (R).
**Figure S4:** Single‐trial analyses of event‐related potentials for correct and erroneous responses.
**Figure S5:** Single‐trial analyses of event‐related potentials for erroneous responses with short and long response durations (RDs).
**Table S1:** Results of cluster‐based permutation tests for differences between short and long response durations (RDs) in lateralized readiness potentials.
**Table S2:** Mean amplitude in the time‐window of the error‐related negativity (ERN), locked to response onset.
**Table S3:** Inferential results for the analyses of mean amplitudes in the time‐window of the error‐related negativity (ERN), locked to response onset.
**Table S4:** Inferential results for the analyses of mean amplitudes in the time‐window of the error‐related negativity (ERN), locked to response onset, separately for correct and erroneous responses (see Table S3 for the full design).
**Table S5:** Comparison of mean amplitudes in the time‐window of the error‐related negativity (ERN) for errors with short versus long response duration (RD), locked to response onset.
**Table S6:** Peak amplitude of the largest negative peak in the time‐window of the error‐related negativity (ERN), locked to response onset.
**Table S7:** Inferential statistics for peak amplitudes of the largest negative peak in the time‐window of the error‐related negativity (ERN), locked to response onset.
**Table S8:** Peak time of the largest negative peak in the time‐window of the error‐related negativity (ERN), locked to response onset.
**Table S9:** Inferential statistics for peak times of the largest negative peak in the time‐window of the error‐related negativity (ERN), locked to response onset.
**Table S10:** Results of cluster‐based permutation tests for differences between errors with short and long response durations (RDs) in event‐related potentials (ERPs), locked to response onset.
**Table S11:** Results of cluster‐based permutation tests for differences between errors with short and long response durations (RDs) in event‐related potentials (ERPs), locked to response offset.
**Table S12:** Results of cluster‐based permutation tests for differences between erroneous and correct responses in a residue‐iteration decomposition (RIDE) of the data.
**Table S13:** Results of cluster‐based permutation tests for differences between errors with short and long response durations (RDs) in a residue‐iteration decomposition (RIDE) of the data.

## Data Availability

Experimental procedures and analyses were preregistered on the Aspredicted platform (https://aspredicted.org/LR9_JJL). Data, codebooks and analysis scripts are available online at https://osf.io/4vncf/?.
