# Supplementary material for: Error‐Related Brain Activity Indicates Immediate Auto‐Cancellation of Action Slips
Source: Psychophysiology. 2025 Oct 7;62(10):e70160. doi: 10.1111/psyp.70160 (PMC12501828; doi:10.1111/psyp.70160)
Supplement: Supplementary file 1 — Figure S1: Lateralized readiness potentials (LRPs), time‐locked to response onset and response offset, respectively. Figure S2: Event‐related potentials (ERPs), locked to response onset (R). Figure S3: Event‐related potentials (ERPs), locked to response offset (R). Figure S4: Single‐trial analyses of event‐related potentials for correct and erroneous responses. Figure S5: Single‐trial analyses of event‐related potentials for erroneous responses with short and long response durations (RDs). Table S1: Results of cluster‐based permutation tests for differences between short and long response durations (RDs) in lateralized readiness potentials. Table S2: Mean amplitude in the time‐window of the error‐related negativity (ERN), locked to response onset. Table S3: Inferential results for the analyses of mean amplitudes in the time‐window of the error‐related negativity (ERN), locked to response onset. Table S4: Inferential results for the analyses of mean amplitudes in the time‐window of the error‐related negativity (ERN), locked to response onset, separately for correct and erroneous responses (see Table S3 for the full design). Table S5: Comparison of mean amplitudes in the time‐window of the error‐related negativity (ERN) for errors with short versus long response duration (RD), locked to response onset. Table S6: Peak amplitude of the largest negative peak in the time‐window of the error‐related negativity (ERN), locked to response onset. Table S7: Inferential statistics for peak amplitudes of the largest negative peak in the time‐window of the error‐related negativity (ERN), locked to response onset. Table S8: Peak time of the largest negative peak in the time‐window of the error‐related negativity (ERN), locked to response onset. Table S9: Inferential statistics for peak times of the largest negative peak in the time‐window of the error‐related negativity (ERN), locked to response onset. Table S10: Results of cluster‐based permutation tests for differences between er [file PSYP-62-e70160-s001.pdf]

Error-related brain activity indicates immediate auto-cancellation of action slips – Supplementary Material

Table of contents:

Supplementary Results

Supplementary References

Figs. S1 to S5

Tables S1 to S13

Supplementary Results

Lateralized readiness potential (LRP) results

We assessed LRPs to probe for signs of differential motor activity related to the execution of an error cancellation movement of withdrawing the finger from the key, specifically for erroneous responses with short RDs. LRPs were computed for electrode sites C3 and C4 using a standard subtraction formula (Eimer, 1998):

$$LRP = \frac{(C3 - C4)_{Left\ Response} - (C3 - C4)_{Right\ Response}}{2}$$

Left and right responses here refer to the actually performed response, not the nominally correct response in case of commission errors so that we expected a positive-going LRP signature before response onset across accuracy conditions and RDs. Validation analyses were run with the electrode pair P3/P4 using the same computation.

To capture systematic LRP differences between short and long RDs (separately for correct and erroneous responses), we performed cluster-based permutation tests (Maris & Oostenveld, 2007). In a first step, we thus performed a two-tailed paired *t*-test for each time point and determined clusters of consecutive significant values. We then summed the resulting *t* values for each cluster (Σt) and determined the probability of these or more extreme sums relative to 100,000 permutations of the data. Figure S1 shows the resulting significant clusters and Table S1 summarizes the corresponding permutation statistics.

The permutation tests showed increased LRP activity that coincided with response offset for short relative to long RDs, suggesting motor activity that aimed at terminating the ongoing movement. This pattern was particularly pronounced for errors, which is consistent with the hypothesized process of actively cancelling ongoing erroneous movements.

ERN results: Locked to response onset

Figure S2 shows the resulting ERPs across the vertex electrodes Fz, FCz, Cz, and Pz. We first verified that all main results replicate when restricting the EEG analysis to erroneous trials with two leading correct trials and two trailing correct trials as used for the behavioral results. This was the case, with larger ERN amplitudes for short as compared to long errors (-4.15 µV vs. -1.42 µV), $t(29) = 2.53$, $p = .017$, $\Delta = 2.73$ µV, 95% $CI_{\Delta} = [0.52$ µV, 4.94 µV$]$, $d_z = 0.46$, 95% $CI_{SM} = [0.08, 0.84]$ at electrode site FCz (with the same overall peak time and thus the same range of [32 ms, 82 ms] as for the main analyses). We still preferred to use the pre-registered selection of all errors with at least one preceding correct trial to enable a maximally large database for all EEG analyses.

Table S2 shows descriptive statistics for the analysis of mean amplitudes, supplemented by ANOVA statistics for the full factorial design in Table S3 and separate ANOVAs for correct and erroneous responses in Table S4. A face-to-face comparison of mean ERN amplitudes for short versus long errors is provided in Table S5. Peak amplitude statistics are shown in Tables S6-S7, and peak times are shown in Tables S8-S9. We further conducted permutation tests as for the LRP data, running separate tests for each electrode. Table S10 shows the corresponding cluster statistics.

ERN results: Locked to response offset

The offset-locked data mirrored the onset-locked pattern perfectly (see Figure S2-S3). Permutation tests were conducted as for the onset-locked ERN data and Table S11 shows the corresponding permutation statistics.

RIDE results

We performed cluster-based permutation tests as above to assess differences between correct and erroneous responses (Figure S4) as well as between errors with short and long RDs (Figure S5), running separate tests for electrodes Fz, FCz, Cz, and Pz for each RIDE cluster (R-Onset, Intermediate, R-Offset). Tables S12-S13 show the corresponding permutation statistics.

Supplementary References

Eimer, M. (1998). The lateralized readiness potential as an on-line measure of central response activation processes. *Behavior Research Methods, Instruments, & Computers*, 30, 146–156. https://doi.org/10.3758/BF03209424

Gehring, W. J., & Fencsik, D. (1999). Slamming on the brakes: An electrophysiological study of errors response inhibition. Poster presented at the *Annual Meeting of the Cognitive Neuroscience Society*, Washington, D. C., 11-13 April 1999.

Maris, E., & Oostenveld, R. (2007). Nonparametric statistical testing of EEG- and MEG-data. *Journal of Neuroscience Methods*, *164*, 177–190. https://doi.org/10.1016/j.jneumeth.2007.03.024

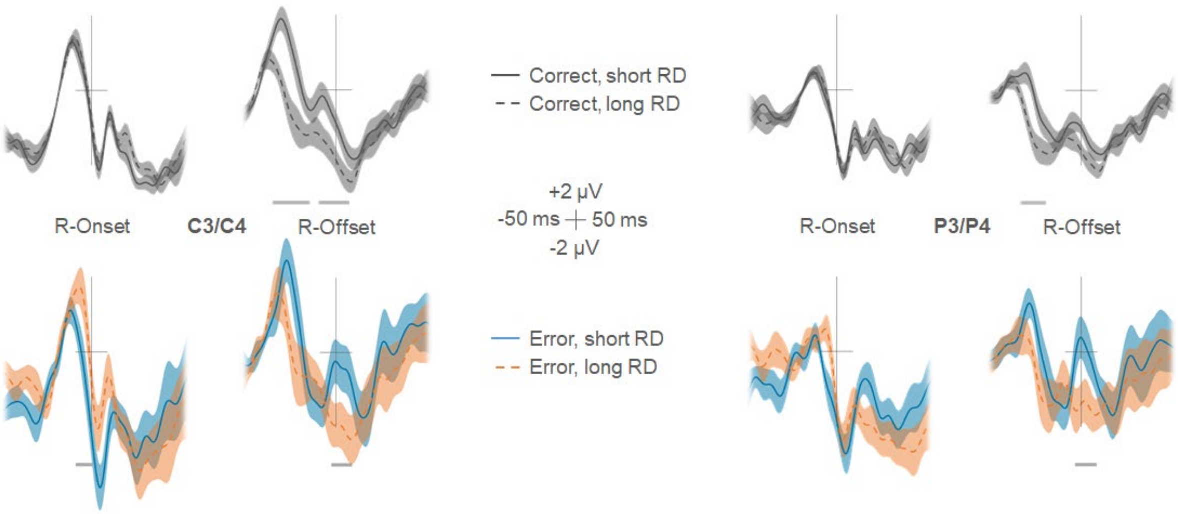

Fig. S1. Lateralized readiness potentials (LRPs), time-locked to response onset and response offset, respectively. Crucially, short errors came with increased LRP activity that coincided with response offset, indicating active cancellation of the erroneous movement. A reduced effect in the same direction was evident for correct responses, possibly indicating cancellation of premature responses. Horizontal bars show significant differences as indicated by permutation tests.

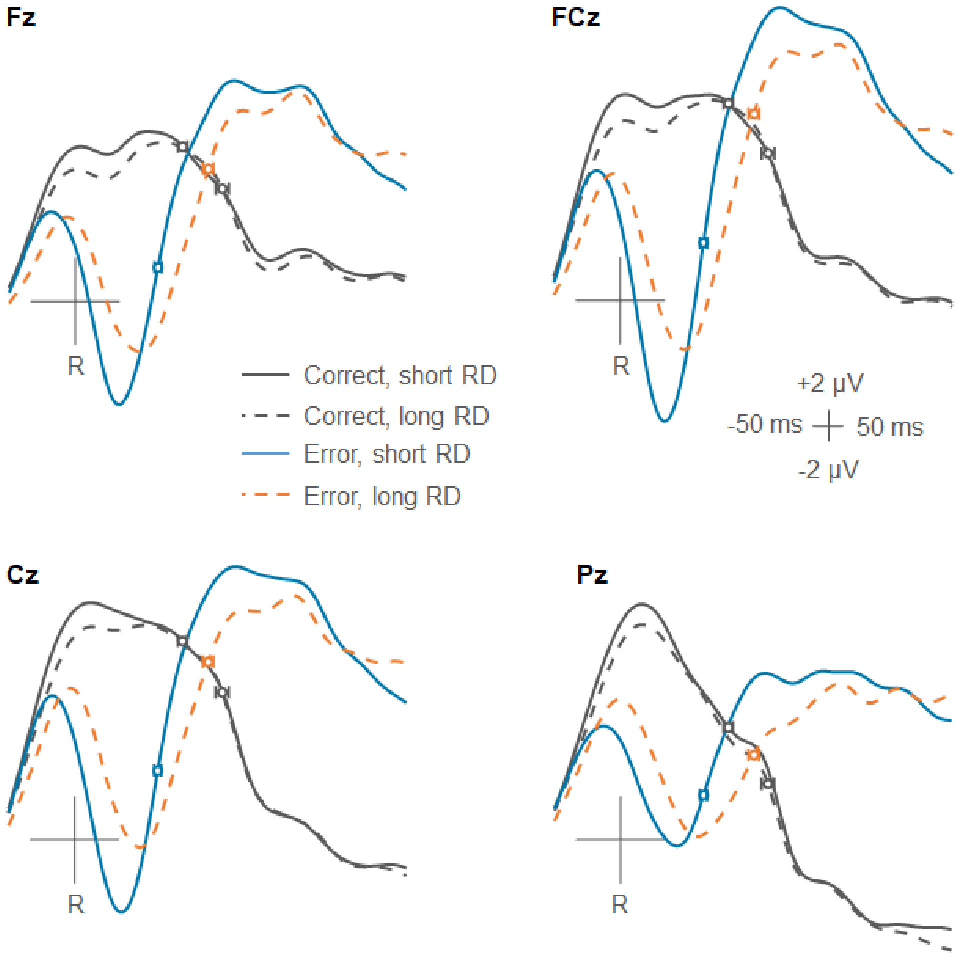

Fig. S2. Event-related potentials (ERPs), locked to response onset (R). ERPs are depicted for correct and erroneous responses with short and long response durations (RDs) for the vertex electrodes Fz, FCz, Cz, and Pz. Response offsets ± standard error shown as point overlays.

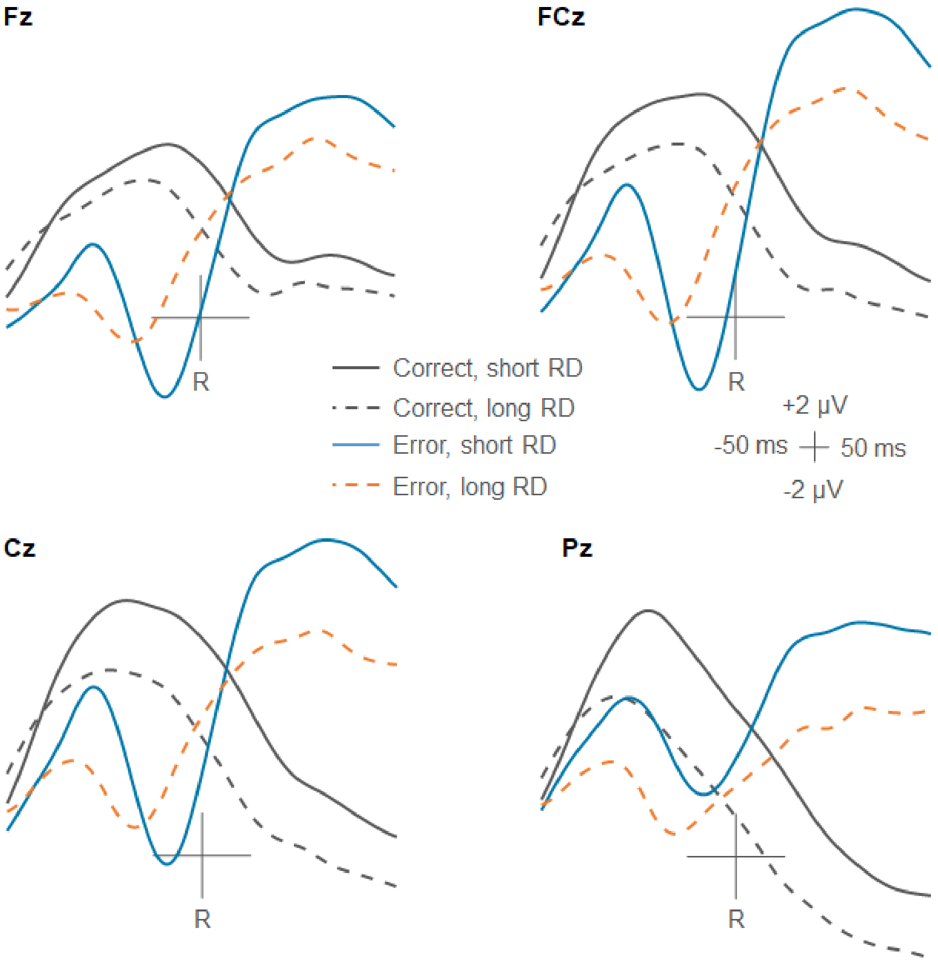

Fig. S3. Event-related potentials (ERPs), locked to response offset (R). ERPs are depicted for correct and erroneous responses with short and long response durations (RDs) for the vertex electrodes Fz, FCz, Cz, and Pz.

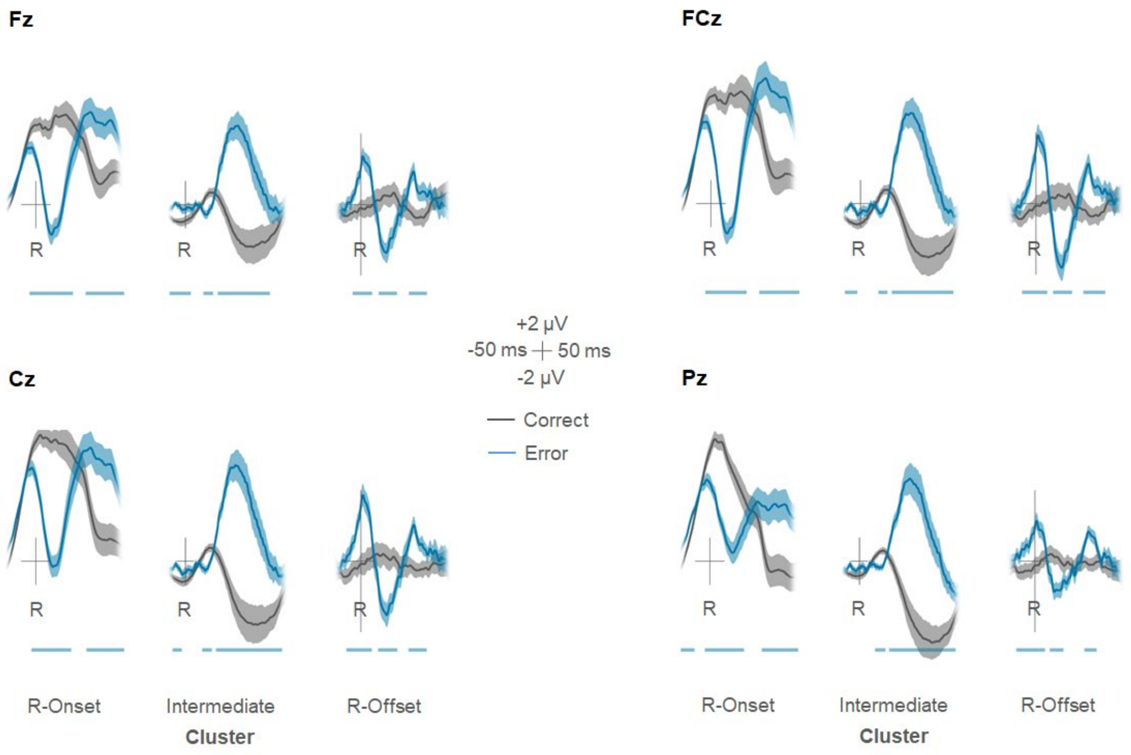

Fig. S4. Single-trial analyses of event-related potentials for correct and erroneous responses. Residue-iteration decomposition (RIDE) into three distinct clusters relative to the response (R) for the vertex electrodes Fz, FCz, Cz, and Pz. Shaded areas indicate standard errors of paired differences between correct and erroneous responses, whereas horizontal bars show significant differences as indicated by permutation tests.

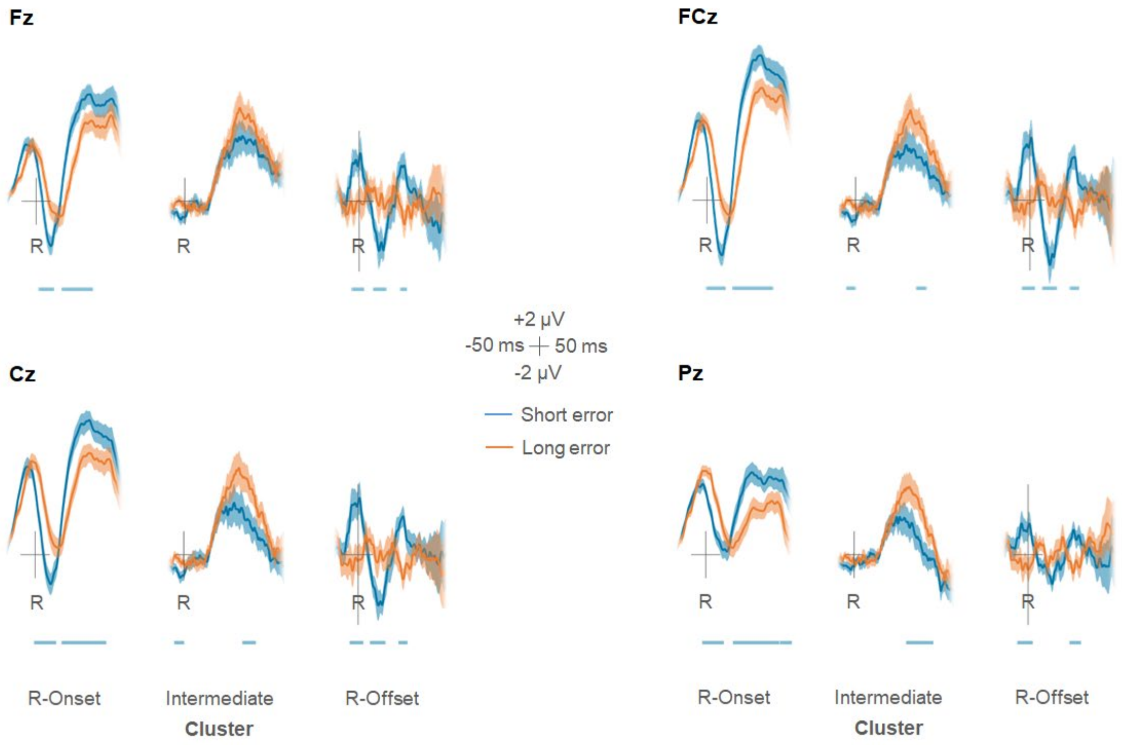

Fig. S5. Single-trial analyses of event-related potentials for erroneous responses with short and long response durations (RDs). Residue-iteration decomposition (RIDE) into three distinct clusters relative to the response (R) for the vertex electrodes Fz, FCz, Cz, and Pz. Shaded areas indicate standard errors of paired differences between short and long RDs, whereas horizontal bars show significant differences as indicated by permutation tests.

Response	Electrode pair	Response event	Permutation clusters			
			Effect direction	Time [ms]	Σt	p
Correct	C3/C4	Offset	Short RD > Long RD	[-196, -81]	419.67	0.001
				[-54, 41]	267.60	0.008
	P3/P4	Offset	Short RD > Long RD	[-187, -109]	252.65	0.004
Error	C3/C4	Onset	Short RD < Long RD	[-49, 2]	-137.77	0.040
		Offset	Short RD > Long RD	[-14, 50]	170.18	0.029
	P3/P4	Offset	Short RD > Long RD	[-19, 48]	181.24	0.022

Table S1. Results of cluster-based permutation tests for differences between short and long response durations (RDs) in lateralized readiness potentials. Clusters of consecutive significant differences were sampled in a search frame of -300 ms to 300 ms relative to the respective response event in lateralized activity at electrode pairs C3/C4 and P3/P4. The summed t values (Σt) of these clusters were tested against 100,000 permutations of the data. The effect direction denotes whether activity was more or less positive for short compared to long RDs.

Electrode	RD	Response	*M* [μV]	Δ (SE_{PD}) [μV]	d_z	BF_{10}
Fz	Short	Correct	7.29	10.92 (0.89)	2.23	1.72E+10
		Error	-3.63			
	Long	Correct	6.35	7.88 (0.84)	1.71	4.46E+07
		Error	-1.53			
FCz	Short	Correct	9.18	13.36 (1.04)	2.35	5.75E+10
		Error	-4.18			
	Long	Correct	8.32	9.48 (0.88)	1.96	8.76E+08
		Error	-1.16			
Cz	Short	Correct	10.44	12.77 (1.09)	2.14	6.84E+09
		Error	-2.34			
	Long	Correct	9.79	9.02 (0.85)	1.93	6.12E+08
		Error	0.77			
Pz	Short	Correct	9.26	9.05 (0.89)	1.86	2.78E+08
		Error	0.21			
	Long	Correct	8.71	6.75 (0.75)	1.65	2.22E+07
		Error	1.96			

Table S2. Mean amplitude in the time-window of the error-related negativity (ERN), locked to response onset. Grand-average means (*M*) along the vertex electrodes, separately for correct and erroneous responses with short and long response durations (RDs). These statistics were evaluated in terms of paired differences in mean amplitudes (Δ) between correct and erroneous responses with standard errors of the paired differences (SE_{PD}), resulting effect size d_z and Bayes Factors (BF_{10}).

Effect	*df*s	*F*	*p*	η_p^2	ε
Intercept	1, 29	72.29	< .001	.71	
Electrode	3, 87	31.64	< .001	.52	0.60
Response	1, 29	170.38	< .001	.85	
Duration	1, 29	5.02	.033	.15	
Electrode × Response	3, 87	33.20	< .001	.53	0.62
Electrode × Duration	3, 87	7.15	.002	.20	0.68
Response × Duration	1, 29	14.36	.001	.33	
Electrode × Response × Duration	3, 87	8.22	.001	.22	0.59

Table S3. Inferential results for the analyses of mean amplitudes in the time-window of the error-related negativity (ERN), locked to response onset. Main effects and interactions in the analysis of variance (ANOVA) on mean amplitudes with the repeated-measures factors electrode (Fz vs. FCz vs. Cz vs. Pz), response duration (RD: short vs. long), and response (correct vs. error). We report ε estimates for Greenhouse-Geisser corrections whenever Mauchly's test indicated violations of the sphericity assumption.

Response	Effect	*df*s	*F*	*p*	η_p^2	ε
Correct	Intercept	1, 29	221.77	< .001	.88	
	Electrode	3, 87	17.49	< .001	.38	0.51
	Duration	1, 29	0.25	.620	.01	
	Electrode × Duration	3, 87	2.60	.088	.08	
Error	Intercept	1, 29	5.40	.027	.16	
	Electrode	3, 87	35.19	< .001	.55	0.71
	Duration	1, 29	4.94	.034	.15	
	Electrode × Duration	3, 87	6.11	.005	.17	0.61

Table S4. Inferential results for the analyses of mean amplitudes in the time-window of the error-related negativity (ERN), locked to response onset, separately for correct and erroneous responses (see Table S3 for the full design). We report ε estimates for Greenhouse-Geisser corrections whenever Mauchly's test indicated violations of the sphericity assumption.

Electrode	RD	Response	M (in µV)	Δ (SE_{PD}; in µV)	d_z	BF_{10}
Fz	Short	Error	-3.63	-2.09 (0.71)	0.54	5.51
	Long		-1.53			
FCz	Short		-4.18	-3.02 (0.83)	0.67	29.99
	Long		-1.16			
Cz	Short		-2.33	-3.10 (0.84)	0.67	31.02
	Long		0.77			
Pz	Short		0.21	-1.74 (0.72)	0.44	1.88
	Long		1.96			

Table S5. Comparison of mean amplitudes in the time-window of the error-related negativity (ERN) for errors with short versus long response duration (RD), locked to response onset. Grand-average mean amplitudes (M) are shown for the vertex electrodes Fz, FCz, Cz, and Pz. These statistics were evaluated in terms of paired differences in mean amplitudes (Δ) between errors with short and long RD with standard errors of the paired differences (SE_{PD}), resulting effect size d_z, and Bayes Factors (BF_{10}).

Electrode	RD	Response	*M* [µV]	Δ (SE_{PD}) [µV]	d_z	BF_{10}
Fz	Short	Error	-5.99	-1.76 (0.64)	0.51	3.81
	Long		-4.22			
FCz	Short		-6.99	-2.90 (0.66)	0.82	187.82
	Long		-4.10			
Cz	Short		-4.86	-2.77 (0.69)	0.75	70.55
	Long		-2.09			
Pz	Short		-1.67	-0.28 (0.66)	0.08	0.16
	Long		-1.39			

Table S6. Peak amplitude of the largest negative peak in the time-window of the error-related negativity (ERN), locked to response onset. The table lists only erroneous trials because correct trials naturally did not produce a pronounced negative peak. Mean peak amplitudes (*M*) for the vertex electrodes Fz, FCz, Cz, and Pz are shown, separately for errors with short and long response durations (RDs). These statistics were evaluated in terms of paired differences in peak amplitudes (Δ) between correct and erroneous responses with standard errors of the paired differences (SE_{PD}), resulting effect size d_z, and Bayes Factors (BF_{10}).

Response	Effect	*df*s	*F*	*p*	η_p^2	ε
Error	Intercept	1, 28	50.41	< .001	.64	
	Electrode	3, 84	32.25	< .001	.54	0.64
	Duration	1, 28	10.24	.003	.27	
	Electrode × Duration	3, 84	14.83	< .001	.35	0.61

Table S7. Inferential statistics for peak amplitudes of the largest negative peak in the time-window of the error-related negativity (ERN), locked to response onset. Main effects and interactions in the analysis of variance (ANOVA) on ERN peak amplitudes with the repeated-measures factors electrode (Fz vs. FCz vs. Cz vs. Pz) and response duration (RD: short vs. long). We report ε estimates for Greenhouse-Geisser corrections whenever Mauchly's test indicated violations of the sphericity assumption.

Electrode	RD	Response	M [ms]	Δ (SE_{PD}) [ms]	d_z	BF_{10}
Fz	Short	Error	53.97	-21.72 (3.10)	1.30	134807.35
	Long		75.69			
FCz	Short		52.45	-22.66 (3.39)	1.24	58922.04
	Long		75.10			
Cz	Short		56.38	-19.10 (3.55)	1.00	2227.44
	Long		75.48			
Pz	Short		67.48	-25.93 (7.35)	0.66	21.50
	Long		93.41			

Table S8. Peak time of the largest negative peak in the time-window of the error-related negativity (ERN), locked to response onset. The table lists only erroneous trials because correct trials naturally did not produce a pronounced negative peak. Mean peak times (*M*) are given for the vertex electrodes Fz, FCz, Cz, and Pz, separately for errors with short and long response durations (RDs). These statistics were evaluated in terms of paired differences in peak amplitudes (Δ) between correct and erroneous responses with standard errors of the paired differences (SE_{PD}), resulting effect size d_z, and Bayes Factors (BF_{10}).

Response	Effect	*df*s	*F*	*p*	η_p^2	ε
Error	Intercept	1, 28	445.75	< .001	.94	
	Electrode	3, 84	22.22	< .001	.44	0.55
	Duration	1, 28	41.09	< .001	.59	
	Electrode × Duration	3, 84	0.61	.486	.02	

Table S9. Inferential statistics for peak times of the largest negative peak in the time-window of the error-related negativity (ERN), locked to response onset. Main effects and interactions in the analysis of variance (ANOVA) on ERN peak amplitudes with the repeated-measures factors electrode (Fz vs. FCz vs. Cz vs. Pz) and response duration (RD: short vs. long). We report ε estimates for Greenhouse-Geisser corrections in case of significant sphericity violations.

Response	Electrode	Response event	Permutation clusters			
			Effect direction	Time [ms]	Σt	*p*
Error	Fz	Onset	Short RD > Long RD	[87, 170]	306.10	0.007
				[-74, -29]	166.69	0.027
			Short RD < Long RD	[-1, 65]	-254.27	0.011
	FCz		Short RD > Long RD	[87, 190]	382.82	0.004
				[-75, -27]	182.49	0.023
			Short RD < Long RD	[-6, 67]	-338.55	0.006
	Cz		Short RD > Long RD	[92, 198]	363.97	0.005
				[-77, -28]	186.06	0.024
			Short RD < Long RD	[-7, 69]	-345.58	0.005
	Pz		Short RD > Long RD	[96, 201]	356.35	0.005
			Short RD < Long RD	[-13, 62]	-303.82	0.008

Table S10. Results of cluster-based permutation tests for differences between errors with short and long response durations (RDs) in event-related potentials (ERPs), locked to response onset. We searched for clusters of consecutive significant differences between short and long RDs in a search window of -100 ms to 300 ms relative to response onset. For the resulting clusters we summed the corresponding *t* values (Σt) and evaluated the cluster statistic against 100,000 permutations of the data. The effect direction denotes whether activity was more or less positive for short compared to long RDs.

Response	Electrode	Response event	Permutation clusters			
			Effect direction	Time segment (in ms)	Σ*t*	*p*
Error	Fz	Offset	Short RD > Long RD	[-122, -77]	131.37	0.041
			Short RD < Long RD	[-55, 19]	-391.37	< 0.001
	FCz		Short RD > Long RD	[-128, -73]	188.75	0.013
				[-298, -253]	123.10	0.048
			Short RD < Long RD	[-57, 15]	-429.10	< 0.001
	Cz		Short RD > Long RD	[-131, -67]	242.26	0.005
				[42, 101]	163.37	0.024
				[-299, -253]	127.23	0.0451
			Short RD < Long RD	[-51, 8]	-282.43	0.002
	Pz		Short RD > Long RD	[-158, -38]	506.70	< 0.001
				[10, 101]	335.74	0.004

Table S11. Results of cluster-based permutation tests for differences between errors with short and long response durations (RDs) in event-related potentials (ERPs), locked to response offset. We searched for clusters of consecutive significant differences between short and long RDs in a search window of -300 ms to 100 ms relative to response onset. For the resulting clusters we summed the corresponding *t* values (Σ*t*) and evaluated the cluster statistic against 100,000 permutations of the data. The effect direction denotes whether activity was more or less positive for short compared to long RDs.

	Electrode	RIDE cluster	Effect direction	Time [ms]	Σ*t*	*p*
			Permutation clusters			
Comparison: correct vs. error (RIDE)	Fz	R-Onset	Correct > Error	[-22, 126]	1052.94	< .001
			Correct < Error	[169, 300]	-463.92	0.004
		Intermediate	Correct > Error	[106, 164]	205.51	0.041
			Correct < Error	[191, 499]	-1733.94	< .001
				[-94, 32]	-457.62	0.014
		R-Offset	Correct > Error	[60, 122]	264.56	< .001
			Correct < Error	[-28, 38]	-273.59	< .001
				[162, 223]	-212.14	0.001
	FCz	R-Onset	Correct > Error	[-19, 121]	1069.79	< .001
			Correct < Error	[164, 301]	-591.26	< .001
		Intermediate	Correct > Error	[106, 159]	195.86	0.044
			Correct < Error	[186, 549]	-2057.07	< .001
				[-92, -19]	-275.95	0.030
		R-Offset	Correct > Error	[58, 122]	282.47	< .001
			Correct < Error	[-47, 38]	-363.60	< .001
				[160, 234]	-264.76	< .001
	Cz	R-Onset	Correct > Error	[-15, 120]	1002.75	< .001
			Correct < Error	[172, 301]	-548.64	0.001
		Intermediate	Correct > Error	[99, 156]	215.99	0.040
				[180, 571]	-2396.04	< .001
				[-76, -22]	-179.12	0.047
		R-Offset	Correct > Error	[58, 123]	268.81	< .001
			Correct < Error	[-49, 37]	-366.62	< .001
				[161, 222]	-229.39	0.001
	Pz	R-Onset	Correct > Error	[-17, 116]	854.60	< .001
			Correct < Error	[176, 301]	-505.43	0.002
				[-99, -52]	-193.29	0.040

Intermediate	Correct > Error	[92, 153]	245.16	0.030
	Correct < Error	[175, 568]	-2529.46	< .001
R-Offset	Correct > Error	[50, 96]	165.43	0.003
	Correct < Error	[-63, 34]	-341.94	< .001
		[167, 208]	-166.00	0.003

Table S12. Results of cluster-based permutation tests for differences between erroneous and correct responses in a residue-iteration decomposition (RIDE) of the data. We applied a search window of -100 ms to 300 ms for the R-Onset cluster and the R-Offset clusters (R = response). The search window was broadened to -100 ms to 600 ms for the Intermediate cluster to capture the extended error positivity emerging in this cluster. For the resulting clusters we summed the corresponding *t* values (Σt) and evaluated the cluster statistic against 100,000 permutations of the data. The effect direction denotes whether activity was more or less positive for correct as compared to erroneous responses.

	Electrode	RIDE cluster	Permutation clusters			
			Effect direction	Time [ms]	Σ*t*	*p*
Comparison: short versus long errors (RIDE)	Fz	R-Onset	Short RD > Long RD	[87, 195]	374.72	0.001
			Short RD < Long RD	[8, 61]	-199.58	0.011
		R-Offset	Short RD > Long RD	[-26, 17]	134.62	0.006
				[139, 163]	85.05	0.028
			Short RD < Long RD	[47, 93]	-138.95	0.006
	FCz	R-Onset	Short RD > Long RD	[87, 227]	491.58	0.001
			Short RD < Long RD	[-3, 65]	-314.00	0.003
		Intermediate	Short RD < Long RD	[-56, 1]	-156.22	0.042
				[359, 423]	-154.04	0.043
		R-Offset	Short RD > Long RD	[-28, 18]	169.23	0.003
			Short RD < Long RD	[135, 167]	132.00	0.009
				[41, 92]	-181.77	0.002
	Cz	R-Onset	Short RD > Long RD	[90, 243]	502.48	< 0.001
			Short RD < Long RD	[-4, 72]	-337.96	0.003
		Intermediate	Short RD < Long RD	[348, 429]	-217.73	0.023
				[-57, 2]	-155.55	0.043
		R-Offset	Short RD > Long RD	[-29, 18]	170.27	0.002
				[137, 168]	137.40	0.006
			Short RD < Long RD	[40, 93]	-173.58	0.002
	Pz	R-Onset	Short RD > Long RD	[93, 252]	511.15	0.001
				[254, 295]	112.57	0.046
			Short RD < Long RD	[-12, 62]	-302.85	0.004
		Intermediate	Short RD < Long RD	[310, 472]	-426.53	0.003
		R-Offset	Short RD > Long RD	[-37, 16]	164.44	0.001
				[141, 180]	130.07	0.004

Table S13. Results of cluster-based permutation tests for differences between errors with short and long response durations (RDs) in a residue-iteration decomposition (RIDE) of the data. We applied a search window of -100 ms to 300 ms for the R-Onset cluster and the R-Offset clusters (R = response). The search window was broadened to -100 ms to 600 ms for the Intermediate cluster to capture the extended error positivity emerging in this cluster. For the resulting clusters we summed the corresponding *t* values (Σt) and evaluated the cluster statistic against 100,000 permutations of the data. The effect direction denotes whether activity was more or less positive for short compared to long RDs.
